# Supplementation of diet with krill oil protects against experimental rheumatoid arthritis

**DOI:** 10.1186/1471-2474-11-136

**Published:** 2010-06-29

**Authors:** Michelle Ierna, Alison Kerr, Hannah Scales, Kjetil Berge, Mikko Griinari

**Affiliations:** 1 MD Biosciences Gmbh Postfach, Gewerbestrasse 9, 8132 Egg b. Zürich, Switzerland; 2 Aker BioMarine ASA, Fjordallèen 16, P.O Box 1423 Vika, NO-0115 Oslo, Norway; 3 Clanet ltd, Kultarinnantie 1b, 02660 Espoo, Finland

## Abstract

**Background:**

Although the efficacy of standard fish oil has been the subject of research in arthritis, the effect of krill oil in this disease has yet to be investigated. The objective of the present study was to evaluate a standardised preparation of krill oil and fish oil in an animal model for arthritis.

**Methods:**

Collagen-induced arthritis susceptible DBA/1 mice were provided *ad libitum *access to a control diet or diets supplemented with either krill oil or fish oil throughout the study. There were 14 mice in each of the 3 treatment groups. The level of EPA + DHA was 0.44 g/100 g in the krill oil diet and 0.47 g/100 g in the fish oil diet. Severity of arthritis was determined using a clinical scoring system. Arthritis joints were analysed by histopathology and graded. Serum samples were obtained at the end of the study and the levels of IL-1α, IL-1β, IL-7, IL-10, IL-12p70, IL-13, IL-15, IL-17 and TGF-β were determined by a Luminex™ assay system.

**Results:**

Consumption of krill oil and supplemented diet significantly reduced the arthritis scores and hind paw swelling when compared to a control diet not supplemented with EPA and DHA. However, the arthritis score during the late phase of the study was only significantly reduced after krill oil administration. Furthermore, mice fed the krill oil diet demonstrated lower infiltration of inflammatory cells into the joint and synovial layer hyperplasia, when compared to control. Inclusion of fish oil and krill oil in the diets led to a significant reduction in hyperplasia and total histology score. Krill oil did not modulate the levels of serum cytokines whereas consumption of fish oil increased the levels of IL-1α and IL-13.

**Conclusions:**

The study suggests that krill oil may be a useful intervention strategy against the clinical and histopathological signs of inflammatory arthritis.

## Background

Arthritis and musculoskeletal disorders are among the most prevalent chronic conditions affecting the US population [1]. Osteoarthritis, also known as osteoarthrosis or degenerative joint disease, is the most common type of arthritis, characterised primarily by cartilage loss and synovitis as a result of the aging process and affects approximately 12.1% of the US population aged 25 years or older [1,2]. Similarly, rheumatoid arthritis (RA) is a major cause of morbidity in the Western world [3]. The disease occurs in 0.5-1% of the worldwide adult population and in 1% of the US adult population [4,5]. In contrast to osteoarthritis, the pathologic feature of RA is an autoimmune disorder characterized by the presence of Rheumatoid factor and anti-citrullinated protein antibodies.

The most commonly used non-clinical model of arthritis is Collagen-Induced Arthritis (CIA) and was first reported by Trentham and colleagues where they induced the disease in rats following a single intradermal injection of type II collagen emulsified in Complete Freund's adjuvant (CFA) [6]. A few years later the same pathology was demonstrated in mice [7]. Immunisation with emulsified type II collagen results in a severe polyarthritis reaction which commences 3-4 weeks after the primary immunization. A secondary challenge with type II collagen alone is often required 21 days following the first challenge to ensure optimal arthritis incidence. Similar to RA, the pathogenesis of CIA is a multistep process driven by major histocompatibility complex restricted T cells that mediate destruction of the joint characterized by fibrin deposition, synovial inflammation, periosteal bone formation, pannus formation and ankylosis of one or more articular joints [6,8].

An autoimmune Th1 environment is crucial in the induction and pathogenesis of both clinical RA and in the CIA experimental model in rodents. IFN-γ is produced very early in lymph nodes following primary immunization with collagen/CFA and declines by the end of the acute phase [9]. TNF-α released by synovial macrophages is critical in the pathogenesis of the disease since TNF-α is found in most biopsies and is a target for many of the biological therapeutic agents currently on the market where it reduces clinical responses in 70% of recipient patients with established RA by attenuating acute phase proteins and IL-6 [4]. Anti-TNF agents can halt the progression of bone and cartilage degradation by suppressing bone-resorbing osteoclasts in joint lesions [10]. However, there are a significant proportion of patients who do not respond to TNF blockade through administration of Infliximab or Etanercept [11]. Furthermore, patients can be rendered susceptible to opportunistic infections such as tuberculosis [4]. Hence, it is vital to identify alternative treatments which could address this unmet clinical need with fewer potential side effects.

The two main categories of polyunsaturated fatty acids (PUFAs) are (n-3) and (n-6). The effect of fish oils on RA has been reviewed in several papers [12-14]. Overall, dietary (n-3) fat has modest therapeutic benefits in several inflammatory diseases such as cardiovascular disease [15], atopic dermatitis [16] and arthritis [17-24]. Two different meta-analyses have been published: one including 9 trials published between 1985 and 1992 [25], and another one describing 10 trials published between 1985 and 2002 [26]. In summary, most of the trials where fish oil was administered to patients with RA demonstrate disease modifying effects, and the reviews and meta-analyses concludes that there is strong evidence that (n-3) PUFAs have some clinical benefits in RA. One of the mechanisms by which inflammation is reduced is proposed to be related to the incorporation of eicosapentaenoic acid (EPA) into phospholipid membrane of macrophages, which results in decreased production of arachidonate products [27].

Krill oil is extracted from Antarctic Krill (*Euphausia Superba*), which is a zooplankton crustacean that is rich in phospholipids [28]. In common to fish oils, krill oil contains a high proportion of (n-3) fatty acids. However, krill oil contains a major part of the (n-3) fatty acids in the form of phospholipids, which makes this oil different from fish oils which contain (n-3) fatty acids in the form of either triacylglycerol or fatty acid ethyl esters (such as Omacor/Lovaza). Phospholipids are the primary structures of human cell membranes and the "gatekeepers" of cells through the regulation of healthy cell membranes. The association between phospholipids and long-chain (n-3) fatty acids might facilitate the passage of fatty acid molecules through the intestinal wall, increasing their bioavailability and ultimately improving the (n-3):(n-6) ratio. Interestingly, a recent study demonstrated that oral phosphatidylcholine pretreatment had beneficial effects on the morphological, functional and microcirculatory characteristics of chronic arthritis [29]. In addition to phospholipids, krill oil also contains the antioxidant astaxanthin, mainly in the ester form [28].

In this study, we wanted to compare and investigate whether the administration of krill oil or fish oil, with comparable levels of EPA and docosahexaenoic acid (DHA), could suppress the development of CIA in the genetically susceptible DBA/1 mouse strain. Effects upon the macroscopic changes to the joint, disease incidence, joint destruction and the systemic levels of important inflammatory mediators responsible for pathology were investigated.

## Methods

### Rats and palatability study

Two groups of 2 month old male Wistar rats (n = 5), initial weight 245 to 254 g, were kept in metabolic cages and fed either a control diet supplemented with rapeseed oil (2.5 g/100 g of diet) or a test diet supplemented with an equal amount of krill oil (Superba™ Krill Oil, Aker BioMarine ASA, Oslo, Norway). Each day the rat weight as well as the amount of food consumed was measured for individual rats.

### Mice

3.5 week old male DBA/1 mice were obtained from Harlan, Israel, and housed within the rodent facility at MD Biosciences, Israel. 42 mice were allowed to acclimatise for two weeks prior to study commencement, during this time they had free access to a standard rodent chow (Teklad) and to drinking water. At study commencement (25 days prior to arthritis induction) the mice were provided *ad libitum *access to control diet or diets supplemented with either fish oil (GC Rieber Oils, AS, Kristiansand, Norway), or krill oil (Superba™ Krill oil, Aker BioMarine ASA, Oslo, Norway). There were 14 mice in each of the 3 treatment groups at the start of the study. 1 mouse in the krill oil group did not recover from the anaesthesia after induction of arthritis as described below (Day 27). Further, a total of 15 mice were culled due to arthritis severity during the study. Among these, 8 belonged to the control group, 2 belonged to the krill oil group and 5 belonged to the fish oil group. These 15 mice were culled at Day 62 or 64.

### Diets

The diets were based on the AIN-93G formulation [30], with substitution of soybean oil with a blend of oils (rapeseed oil, sunflower oil, coconut oil and linseed oil). This substitution allowed the 3 diets to be similar for total fatty acids, and for oleic, linoleic and alpha linolenic acids. Fish oil and krill oil diets were further balanced for EPA and DHA content. The 3 diets were prepared by Altromin GmbH & Co. KG (Lage, Germany) and stored in vacuum bags to reduce (n-3) PUFA oxidation. The fatty acid composition of the diets is presented in Table 1. The contents of EPA + DHA were comparable between the two treatments (0.44 g/100 g of krill oil diet and 0.47 g/100 g of fish oil diet). This level of EPA + DHA in the diets is equivalent to 0.8 en%, which was chosen to provide a level of (n-3) PUFA intake achievable in humans, and corresponds to 1.8 g d^-1 ^on a 8400 kJ diet in humans. Fresh food was supplied daily throughout the study. Mice continued to have free access to drinking water. All animal experiment procedures were performed according to the guidelines and protocols approved by the Committee for Ethical Conduct in the Care and Use of Laboratory Animals in Israel.

**Table 1:** Dietary fatty acid composition

Fatty acid	Control	Fish Oil	Krill Oil
	g/100 g diet		
**18:3 (n-3)**	0.26	0.26	0.29

**18:4 (n-3)**	-	0.05	0.08

**20:5 (n-3**	-	0.29	0.30

**22:6 (n-3)**	-	0.18	0.14

Σ **n-3**	0.26	0.78	0.81

**18:2 (n-6)**	2.07	2.23	2.22

**20:4 (n-6)**	-	0.01	0.01

Σ **n-6**	2.07	2.24	2.23

**(n-6)/(n-3)**	7.85	2.86	2.76

**18:1 (n-9)**	2.34	2.72	2.25

Σ **unsaturated**	4.68	5.74	5.29

**12:0**	1.09	0.04	0.02

**14:0**	0.39	0.20	0.38

**16:0**	0.58	0.78	1.09

**18:0**	0.22	0.21	0.19

**20:0**	0.03	0.04	0.03

Σ **saturated**	2.32	1.26	1.71

**Unsaturated/saturated**	2.02	4.54	3.10

### Body weight following arthritis induction

Body weights were determined on day 0 and twice weekly thereafter throughout the study.

### Induction of arthritis

Following 25 days of feeding with the supplemented diets, arthritis was induced in all mice by the intradermal injection of bovine type II collagen (200 μg/mouse) (MD Biosciences, Minneapolis) emulsified in complete Freund's adjuvant (MD Biosciences) at the base of the tail under anaesthesia. Twenty-two days later (study day 47) mice were boosted with an intraperitoneal injection of bovine type II collagen (200 μg/mouse).

### Clinical arthritis assessment

Following the intraperitoneal collagen boost on day 47 clinical signs of arthritis were assessed in all paws three times weekly according to a 0-4 scale of ascending severity. Briefly, grade 0 indicated no swelling or erythema; grade 1, mild, but definite redness and swelling of the ankle/wrist or apparent redness and swelling limited to individual digits, no matter the number of affected digits; grade 2, moderate to severe redness and swelling of the ankle/wrist; grade 3, redness and swelling of the entire paw including digits; grade 4 maximally inflamed limb with involvement of multiple joints. Thus the maximum score for four fully inflamed paws would be 16. Paw thickness as a measure of swelling was determined in mm three times weekly using dial calipers (Kroeplin, Munich, Germany).

### Histopathological assessment of arthritis

At termination, the mouse paws were cut using scissors above the knee joint and fixed in 10% formalin for at least one week. The tissues were then decalcified, trimmed and the knee joints embedded in paraffin. A 6 μm section was cut and stained with haematoxylin and eosin (H&E). The stained knee sections were examined and scored for the parameters, inflammatory infiltrate, synovial hyperplasia and the erosion of cartilage and bone as described in Table 2. Where an animal was found to be intermediate between a score (e.g. between a 1 and 2) then an intermediate score was assigned (e.g. 1.5). This scoring was blinded, with the scorer unaware of the identity of the sample. Subsequent to the scoring the samples were un-blinded for data analysis.

**Table 2:** Histopathological scoring of joint sections

Parameter	Score
*Infiltrate*	

No infiltrate detected.	0

Modest leukocyte infiltrate in synovial tissue, no fluid leukocytes.	1

Moderate leukocyte infiltrate in synovial tissue and in fluid phase with loss of synovial architecture.	2

Gross leukocyte infiltrate in synovial membrane and fluid space with significant loss of synovial and articular architecture.	3
	
*Hyperplasia*	

No abnormalities detected.	0

Synovial lining layer 2-4 cells thick.	1

Synovial lining layer >5 cells thick associated with moderate expansion of the sub lining layer zone.	2

Synovial lining layer >5 cells thick associated with significant expansion of the sub lining layer zone and potentially with loss of synovial architecture.	3
	
*Erosion of cartilage/bone*	

No abnormalities detected.	0

Fibrillation of cartilage and/or mild erosive infiltration of periosteal and subchondral bone. Nuclei intact within lacunae.	1

Moderate fibrillation and loss of cartilage and/or moderate erosive infiltration of periosteal and subchondral bone. Nuclei may show apoptosis within lacunae.	2

Significant loss of cartilage and/or erosive infiltration of periosteal and subchondral bone. Nuclei show apoptosis within lacunae across a wide area of cartilage/bone.	3

### Assessment of systemic cytokine levels

Upon termination of the study blood samples were taken and serum prepared from all surviving mice. Blood was obtained directly by cardiac puncture, and the collected volume was the maximum amount obtainable. Serum samples were stored at -20°C. A blood sample was also taken from any animals culled due to severity of arthritis. The levels of IL-1α, IL-1β, IL-7, IL-10, IL-12p70, IL-13, IL-15, IL-17 and TGF-β were then assessed using Luminex™ multiplex reagents according to the manufacturer's instructions (Millipore, St Charles, MO). For measurement of interleukins we used a Mouse Cytokine/Chemokine Panel, 8-plex (Millipore, cat. no. MPXMCYTO-70K-08). Due to reagent incompatibilities, TGF-β was assayed separately from the other cytokines. For measurement of TGF-β, TGF-beta1, 1-plex (Millipore, cat. no. TGFB-64K-01) was used. Data were collected using a Luminex 100 (Luminex Corporation, Austin, TX). The lowest limit of detection was 5.1 pg/ml for IL-1α, 2 pg/ml for IL-1β, 0.9 pg/ml for IL-7, 3.3 pg/ml for IL-10, 4.1 pg/ml for IL-12p70, 6.3 pg/ml for IL-13, 6.5 pg/ml for IL-15, 0.5 pg/ml for IL-17 and 10 pg/ml for TGF-β. Standard curves were generated using a 5-parameter logistic curve-fitting equation weighted by 1/y (StarStation V 2.0; Applied Cytometry Systems, Sacramento, CA). Each sample reading was interpolated from the appropriate standard curve.

### Fatty acid analysis

The fatty acid composition [31] and the method of fatty acid analysis [32] of the diets have been previously described.

### Statistical analysis

Where appropriate data are expressed as the group mean ± standard error of the mean (SEM). The statistical significances of any treatments was determined by ANOVA with Tukey post hoc testing (arthritis scores, paw thickness, body weight and histology scores) or Mann Whitney U test (palatability data and cytokine levels) using the JMP 8.0 (SAS Institute Inc., Cary, NC). A p-value of less than 0.05 was taken to be significant. Correlations between the arthritis score and body weight at the time of primary immunization with Type II Collagen was carried out using the Spearman Rank Correlation Analysis in Winstat™ add-in for Excel™. A p-value of less than 0.05 was deemed to be significant.

## Results

### Dietary supplementation with krill oil reduces clinical arthritis signs in mice

An initial palatability study was carried out in rats to ensure that the addition of krill oil to the feed did not affect the consumption of food. Rats fed on either rapeseed oil or krill oil supplemented diet gained weight in a similar fashion over the 16 days and consumed similar amount of chow (data not shown). Starting weights were 247.6 ± 0.87 g for rapeseed oil supplemented controls and 251.2 ± 1.53 g for rats fed the krill oil supplemented diet. Thus, it was concluded that dietary supplementation with krill oil does not affect palatability of the feed.

**Figure 1:**
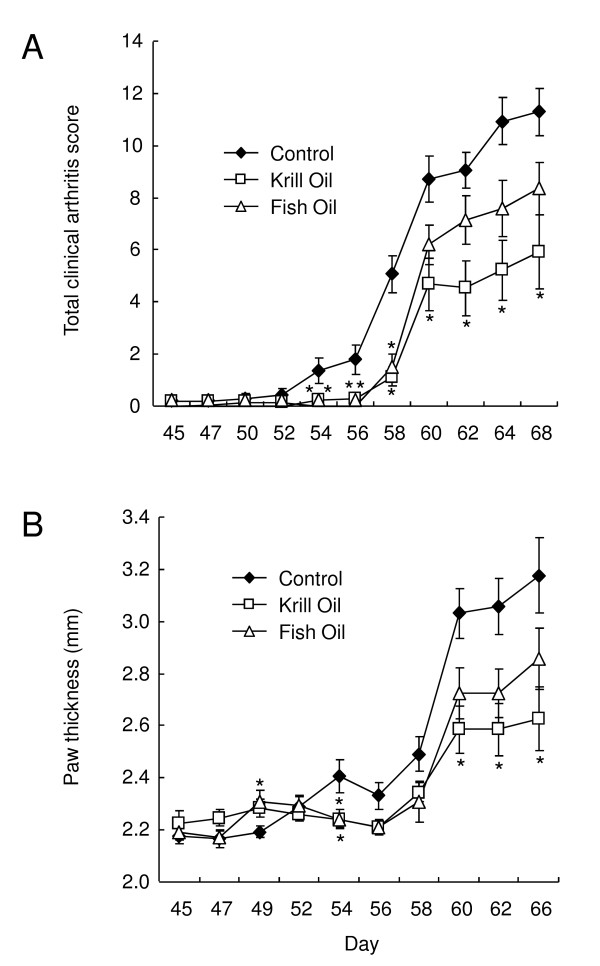
**Krill oil and fish oil reduce clinical arthritis signs in mice**. A) Clinical arthritis scores in DBA/1 mice following induction of collagen induced arthritis with collagen boost on study day 47. B) Hind paw thickness (mm) measurements in DBA/1 mice following induction of collagen induced arthritis on study day 47. Data represents mean values ± SEM (n = 13-14). For mice that were culled due to arthritis severity, the values for the last measurement of clinical arthritis scores and hind paw thickness were used for calculations throughout the rest of the study. * Significantly different from control group (P < 0.05).

As illustrated in Figure 1, arthritis was induced in male DBA/1 mice that had been fed on either control, fish oil supplemented or krill oil supplemented diets for 25 days, by an intradermal injection of bovine type II collagen in CFA followed 22 days later by an intraperitoneal injection of collagen (collagen boost, study day 47). Feeding with the supplemented and control diets was continued throughout the study until day 68.

Clinical arthritis was first noted in the mice fed the control diet on day 45 (prior to the collagen boost, Table 3), and the incidence of disease steadily increased reaching 100% on day 60. Externally, the animals' joints appeared reddened and swollen, with the mean clinical arthritis score reaching 11.29 ± 0.9 (out of a maximum of 16, Figure 1A) on day 68. 8 animals within this group were culled due to welfare as disease was deemed to be too severe, leaving 6 survivors at the end of the study.

**Table 3:** Clinical arthritis scores and arthritis incidence (%) in DBA/1 mice following induction of collagen-induced arthritis with collagen boost on study day 47

		Day										
		45	47	50	52	54	56	58	60	62	64	68
Control	Mean Clinical Arthritis Score	0.21	0.21	0.29	0.43	1.36	1.79	5.07	8.71	9.07	10.93	11.29
	SEM	0.15	0.15	0.22	0.25	0.48	0.58	0.71	0.86	0.69	0.92	0.90
	% Arthritis Incidence	14.3	14.3	14.3	21.4	42.9	57.1	92.9	100	100	100	100
	n =	14	14	14	14	14	14	14	14	14	14	14
	Number of survivors	14	14	14	14	14	14	14	14	14	6	6

Fish Oil	Mean Clinical Arthritis Score	0	0.07	0.14	0.14	0	0	1.50	6.21	7.14	7.57	8.36
	SEM	0	0.07	0.10	0.10	0	0	0.51	0.76	0.92	1.08	1.01
	% Arthritis Incidence	0	7.1	14.3	14.3	0	0	57.1	100	100	100	100
	n =	14	14	14	14	14	14	14	14	14	14	14
	Number of survivors	14	14	14	14	14	14	14	14	11	9	9

Krill Oil	Mean Clinical Arthritis Score	0	0	0	0	0.23	0.31	1.15	4.69	4.54	5.23	5.92
	SEM	0	0	0	0	0.17	0.24	0.39	1.00	1.04	1.15	1.44
	% Arthritis Incidence	0	0	0	0	15.4	15.4	46.2	76.9	76.9	84.6	76.9
	n =	13	13	13	13	13	13	13	13	13	13	13
	Number of survivors	13	13	13	13	13	13	13	13	13	11	11

In the animals fed diets supplemented with either fish oil or krill oil, disease incidence increased more slowly and was not observed at all in the mice fed krill oil supplemented diet until day 54 when it was only 15.4%, compared to the mice fed the control diet where the incidence was 42.9%. This is consistent with the clinical arthritis scoring, which was found to be significantly lower in mice fed the diet supplemented with krill oil compared to that observed in mice fed control diet on study days 54-68. Arthritis score in fish oil supplemented mice were significantly lower than control at day 54, 56 and 58. Furthermore, at the end of the study there were a total of 11 survivors in the group fed with krill oil in comparison to 9 survivors in the group fed with fish oil.

### Dietary supplementation with krill oil reduces hind paw thickness in mice

The extent of hind paw swelling was assessed using a dial caliper (Figure 1B). Hind paw swelling was significantly lower in krill oil supplemented mice at day 54, 60, 62 and 66, compared to control mice. Paw thickness in mice administered fish oil was different from control animals at day 49 and 54. In all groups, the main increase in hind paw swelling occurred after day 58.

### Dietary supplementation with krill oil reduces histopathology associated with arthritis in mice

**Figure 2:**
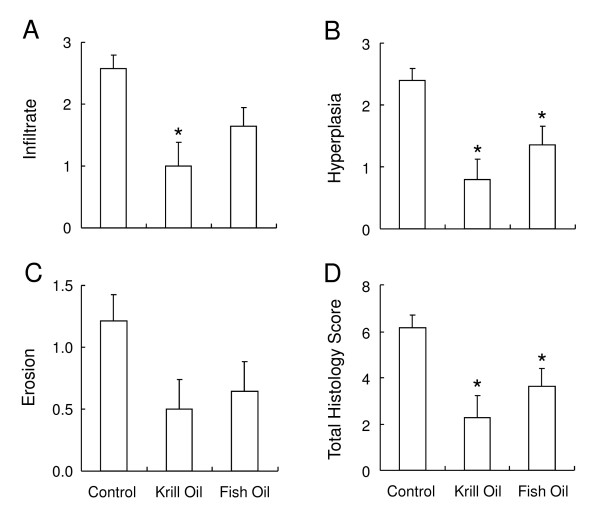
**Krill oil and fish oil reduce histopathology associated with arthritis in mice**. Histopathology scores in sections taken from knee joints of arthritis induced DBA/1 mice on termination of the study. Sections were scored for cell infiltrations (A), synovial hyperplasia (B), bone/cartilage erosion (C) and total histology score (D). Data represents mean values ± SEM (n = 13-14). * Significantly different from control group (P < 0.05).

At termination the rear paws were removed and prepared for histopathological analysis. The knee joints were examined microscopically and scored for signs of inflammatory cell infiltrate (Figure 2A), hyperplasia of the synovial membrane (Figure 2B), erosion of the bone and cartilage (Figure 2C) and total histology score (Figure 2D). In the knees of arthritic mice fed the control diet, inflammatory infiltrate, synovial hyperplasia and bone/cartilage erosion were observed with mean scores of 2.57 ± 0.22, 2.39 ± 0.19 and 1.21 ± 0.21, respectively. Mice fed the supplemented diets showed reduced histopathological scores for all the variables measured, in particular in mice fed the krill oil supplemented diet, which had significantly lower joint infiltrates (1 ± 0.39) and hyperplasia scores (0.70 ± 0.32). Fish oil supplementation led to a significantly altered synovial hyperplasia score (1.36 ± 0.3) only. Total scores were significantly reduced in mice fed both krill oil (2.29 ± 0.94) and fish oil supplemented diets (3.64 ± 0.77) compared to mice fed the control diet (6.18 ± 0.54).

### Dietary supplementation with krill oil increases weight gain

**Figure 3:**
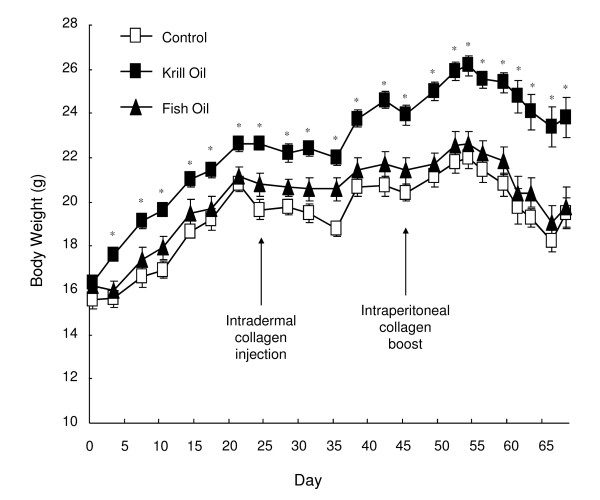
**Effect of krill oil and fish oil on body weight changes in mice**. Mean body weights of DBA/1 mice following induction of collagen induced arthritis with collagen boost on study day 47. Data represents mean values of living mice ± SEM. * Significantly different from control group (P < 0.05).

Body weight was measured twice weekly throughout the study and weight increased in all treatment groups until day 54 (Figure 3). After day 54, the weight decreased in all three treatment groups. This drop in body weight coincided with the development of more severe arthritis (Figure 1A). In mice fed krill oil supplemented diet, weight increase started 2 days earlier than in the other treatment groups although the rate of weight increase appeared to be similar in all groups. This resulted in a significantly higher mean body weight in krill oil fed mice from day 2 until study termination on day 68.

### Dietary supplementation with krill oil does not affect serum cytokines in arthritic mice

**Figure 4:**
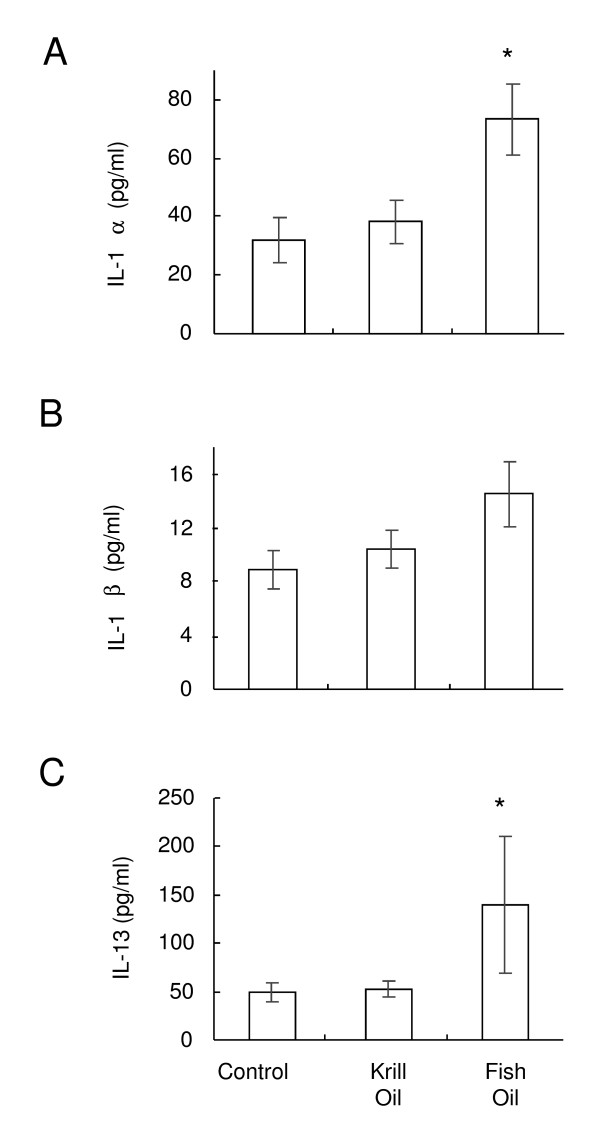
**Effect of krill oil and fish oil on serum cytokine levels**. Level of IL-1α (A), IL-1β (B) and IL-13 (C) detected in the serum from CIA mice on termination of the study. Data represents mean values ± SEM (n = 6-11). * Significantly different from control group (P < 0.05).

The levels of systemic IL-1α, IL-1β, IL-7, IL-10, IL-12p70, IL-13, IL-15, IL-17 and TGF-β were analysed in the serum of mice upon termination. Low baseline levels of IL-7, IL-10, IL-12p70, TGF-β and IL-17 were found in all mice (data not shown). Higher levels of IL-1α, IL-1β and IL-13 were found in the serum from CIA mice (Figure 4). Levels of IL-1α in mice fed fish oil were significantly higher than in mice fed control or krill oil diet (Figure 4A). The IL-1β level tended to increase after fish oil administration, but was not significant (Figure 4B) (*P *= 0.086). The serum level of IL-13 was significantly elevated after treatment with fish oil, but not krill oil (Figure 4C). Between the treatment groups, there were no significant differences between the other cytokines investigated (data not shown).

## Discussion

It has been known for many years that fish oil is able to benefit the joint pain associated with RA [12-14]. In this study, a CIA model was used to compare the effects of krill oil and fish oil on the development of RA. Indeed, the results of this study clearly demonstrate that supplementation of a diet with krill oil is able to significantly inhibit the development of arthritis in the CIA model. This is the first report of krill oil in this or any other experimental animal model of arthritis. At some time points, fish oil also demonstrated a reduction in clinical arthritis score.

The initial palatability study indicated that the krill oil supplemented diet would not affect body weight of animals. During the CIA study, mice fed the krill oil diet had significantly higher body weights from day 3 of the study. In order to explain why krill oil fed mice did not develop arthritis to the same extent as control mice, we performed statistical analysis to explore whether mice with a higher body weight at the time of the collagen boost correlated with a lower arthritis score at the end of the study. The Spearman Rank Correlation Analysis revealed that there was no significant correlation between individual body weights on day 45 (just prior to arthritis boost when krill oil supplemented mice already had significantly higher body weights but before development of significant arthritis) and the final arthritis score for individual mice. Therefore, it is less likely that the ability of krill oil to significantly reduce arthritis severity was simply a result of altered body weight prior to development of arthritis. The increase in body weight after krill oil supplementation is inconsistent with data from another study in mice [33]. In this study, however, mice were administered a high fat diet and the two models are therefore not directly comparable. In addition, data from a toxicology study with krill oil in Wistar rats do not show any significant weight gain after 13 weeks of feeding (data not published).

We also investigated whether the expression of several pro-inflammatory cytokines were altered in mice fed the krill oil or fish oil supplemented feeds. Since pro-inflammatory cytokines such as IL-1, TNF-α and IL-17 are known to drive the joint pathology in RA by activating inflammatory cells and inducing the release of other mediators such as MMPs and causing osteolysis [34], we investigated whether treatment with either fish oil or krill oil supplemented diets would significantly alter the levels of a range of pro- and anti-inflammatory cytokines. We did not find any association between cytokine levels and severity of the disease. Neither of the supplemented diets was able to reduce the expression of pro-inflammatory cytokines. In fact we found that fish oil actually slightly increased the amounts of IL-1α and IL-13 in the serum of mice. It may be that fish oil is able to alter the cytokine milieu towards a more Th2 profile, and hence we saw a 2-fold increase in IL-13 levels. However, the majority of cytokine data found in this study would suggest that systemic cytokines are not good biomarkers for the severity of the study. Future studies would seek to investigate cytokine mRNA expression directly within the joint.

Moving into the joint rather than the circulation, it is known that the pathology seen in the CIA model is similar to that in human RA [6] with inflammatory cell influx, synovial hyperplasia, pannus formation and cartilage/bone erosion.

It has previously been shown that diets rich in fish oil are able to benefit the pathology detected in osteoarthritis susceptible mouse strains [35]. Mice fed fish oil supplemented diets had reduced synovitis when compared to corn oil supplemented diets. We have expanded this information on osteoarthritis to include proof that both krill oil and fish oil can protect against the joint damage seen in RA. In this study, supplementation of the diet with krill oil significantly reduced the magnitude of cell influx, significantly reduced the thickening of the synovial membrane and reduced the cartilage erosion seen in control animals. Krill oil exerted these effects at a level greater than that seen with the fish oil and was the only supplemented diet able to reduce inflammatory cell infiltration into the joint.

In the present study, krill oil was able to reduce the severity of arthritis by about 50%. To put these results with krill oil into context, there have been several investigations with other dietary supplements in the robust CIA model. In a recent publication it was investigated how the severity of CIA was affected in mice given a diet supplemented with conjugated linoleic acid (CLA) [36]. CLA was able to slightly delay the onset of arthritis but did not result in significant reduction in arthritis scores. Hence any effect that CLA may have is mild to moderate in comparison to that exerted by krill oil. Even with such a mild effect seen with CLA supplementation, there is still quite widespread interest in using CLA as a supplement [37]. In a previous study in a CIA model, the effect of fish oil was compared with corn oil [38]. The researchers found that fish oil delayed the onset (34 d compared with 25 d) and reduced the incidence (69% compared with 93%) and severity (6.7 compared with 9.8) of CIA. This study was, however, not directly comparable with our study since they had different mice strain (B10 mice) and higher level of fish oil included in the diet (approximately 3 times higher level of EPA and DHA). However, the study supports our findings that oils rich in (n-3) have beneficial effects in experimental models of arthritis.

One factor that differentiates krill oil from fish oil is the presence of phospholipids in krill oil. More than 40% of the oil consists of phospholipids, and the majority of these phospholipids contain (n-3) fatty acids (data to be published). Unfortunately, little scientific attention has been paid to the absorption and distribution of dietary phospholipids, and almost exclusively, all studies on (n-3) fatty acids in human and experimental animal models have been performed using such fatty acids in either triacylglycerol form, free fatty acid form, or as fatty acid ethyl esters. There is some evidence, however, that the absorption and the distribution of fatty acids from dietary phospholipids can be different than the corresponding fatty acids from triacylglycerol [39-41]. Fatty acids from dietary phospholipids have been reported to be taken up in the brain 2.1-fold more efficiently than fatty acids from triacylglycerol [42]. It has been reported a difference in the pharmacokinetic properties between fatty acids originating from phospholipids versus triacylglycerol [43], and the absorption, intestinal metabolism and distribution of (n-3) fatty acids from dietary phospholipids might be different than (n-3) fatty acids from triacylglycerol. Indeed, in Zucker rats, EPA and DHA from krill oil were more efficiently incorporated into heart phospholipids, compared to EPA and DHA from fish oil [31]. Moreover, a higher incorporation of DHA into brain was also reported after krill oil administration to Zucker rats [44]. Whether this is relevant for the effects observed in the current study is not known, but one possibility is that (n-3) fatty acids from krill oil and fish oil have different effects on inflammation. These mechanisms are only left to speculations, but can for example be related to a different uptake of (n-3) fatty acids in neutrophils and, consequently, an altered lipid composition and function of neutrophils [45]. The presence of EPA, DHA and arachidonic acid in neutrophil phospholipids after krill oil and fish oil administration should be investigated in future studies.

Another difference between krill oil and fish oil is the presence of astaxanthin in krill oil. This antioxidant might be able to exert anti-inflammatory effects and has been found to protect against cardiovascular disease [46]. It is interesting to note that administration of antioxidants such as anthocyanins and edaravone has been shown to have beneficial effects on RA [47,48].

## Conclusions

In conclusion, krill oil provides protection in terms of arthritis scores and joint pathology in the CIA model. Thus, this source of (n-3) fatty acids deserves more investigation as a food supplement for patients suffering from not only RA but also osteoarthritis and other inflammatory conditions.

## Competing interests

KB is employed in Aker BioMarine ASA. MG was a consultant in Aker BioMarine when the experiments were planned and performed. MI, HS and AK were employed at MD Biosciences when the study was performed.

## Authors' contributions

MG designed the study in co-operation with researchers at MD Biosciences. Most of the experimental procedures were carried out by employees at MD Biosciences. MI, HS and AK analysed the data. MI, MG and KB wrote the manuscript. All authors read and approved the final manuscript.

## Pre-publication history

The pre-publication history for this paper can be accessed here:

http://www.biomedcentral.com/1471-2474/11/136/prepub
